# Oura Ring as a Tool for Ovulation Detection: Validation Analysis

**DOI:** 10.2196/60667

**Published:** 2025-01-31

**Authors:** Nina Thigpen, Shyamal Patel, Xi Zhang

**Affiliations:** 1 Oura Ring San Francisco, CA United States

**Keywords:** ovulation, digital medicine, physiology, body temperature, menstrual cycles, wearable, fertility, nonhormonal contraception, reproductive health, women’s health, calendar method, mHealth, mobile health, detection

## Abstract

**Background:**

Oura Ring is a wearable device that estimates ovulation dates using physiology data recorded from the finger. Estimating the ovulation date can aid fertility management for conception or nonhormonal contraception and provides insights into follicular and luteal phase lengths. Across the reproductive lifespan, changes in these phase lengths can serve as a biomarker for reproductive health.

**Objective:**

We assessed the strengths, weaknesses, and limitations of using physiology from the Oura Ring to estimate the ovulation date. We compared performance across cycle length, cycle variability, and participant age. In each subgroup, we compared the algorithm’s performance with the traditional calendar method, which estimates the ovulation date based on an individual’s last period start date and average menstrual cycle length.

**Methods:**

The study sample contained 1155 ovulatory menstrual cycles from 964 participants recruited from the Oura Ring commercial database. Ovulation prediction kits served as a benchmark to evaluate the performance. The Fisher test was used to determine an odds ratio to assess if ovulation detection rate significantly differed between methods or subgroups. The Mann-Whitney *U* test was used to determine if the accuracy of the estimated ovulation date differed between the estimated and reference ovulation dates.

**Results:**

The physiology method detected 1113 (96.4%) of 1155 ovulations with an average error of 1.26 days, which was significantly lower (*U*=904942.0, *P*<.001) than the calendar method’s average error of 3.44 days. The physiology method had significantly better accuracy across all cycle lengths, cycle variability groups, and age groups compared with the calendar method (*P*<.001). The physiology method detected fewer ovulations in short cycles (odds ratio 3.56, 95% CI 1.65-8.06; *P*=.008) but did not differ between typical and long or abnormally long cycles. Abnormally long cycle lengths were associated with decreased accuracy (*U*=22,383, *P*=.03), with a mean absolute error of 1.7 (SEM .09) days compared with 1.18 (SEM .02) days. The physiology method was not associated with differences in accuracy across age or typical cycle variability, while the calendar method performed significantly worse in participants with irregular cycles (*U*=21,643, *P*<.001).

**Conclusions:**

The physiology method demonstrated superior accuracy over the calendar method, with approximately 3-fold improvement. Calendar-based fertility tracking could be used as a backup in cases of insufficient physiology data but should be used with caution, particularly for individuals with irregular menstrual cycles. Our analyses suggest the physiology method can reliably estimate ovulation dates for adults aged 18-52 years, across a variety of cycle lengths, and in users with regular or irregular cycles. This method may be used as a tool to improve fertile window estimation, which can aid in conceiving or preventing pregnancies. This method also offers a low-effort solution for follicular and luteal phase length tracking, which are key biomarkers for reproductive health.

## Introduction

With advances in wearable technology, an increasing number of women are leveraging continuous, automated sensor data to track ovulation [1]. Ovulation tracking serves multiple purposes; notably, it can be used to delineate the end of the fertile window to optimize the timing of intercourse for conception or contraception. Studies have shown that ovulation tracking can increase the likelihood of conception by approximately 40% for those trying to conceive [2-5]. Conversely, fertility awareness–based methods for contraception, although less effective than hormonal contraception methods [6-8], is increasing popular as the use of hormonal contraception declines [9-12]. Beyond fertility, the ovulation date, alongside period start dates, can be used to measure a cycle’s follicular and luteal lengths. These phase lengths have been identified as biomarkers of menstrual health [13], with early research suggesting they may provide insights into fecundity [14], systemic inflammation [15], endometrial development [16], and ovarian aging [17].

Approximately 75% of individuals attempting to conceive use some method to estimate their ovulation date; these methods include cervical mucus monitoring, detecting a rise in basal body temperature (BBT), and feeling for changes in the cervix [18]. These methods vary in accuracy across subgroups including cycle regularity, compliance, and interpretation [19]. Among these, cervical mucus tracking is associated with the highest reported accuracy, with 48% to 76% of ovulations correctly estimated within 1 day of the reference ovulation [19,20]. Approximately 21% of women trying to conceive use the BBT method [18], which, although less precise than cervical mucus tracking at predicting imminent ovulation, has been found to accurately detect that ovulation has occurred [21-23]. All these methods—cervical mucus tracking, feeling for changes in the cervix, and BBT—require significant user knowledge and active participation. In contrast, wearable-based ovulation tracking may provide a more convenient alternative for those seeking a less labor-intensive approach to monitoring ovulation [1].

Previous research has shown that wearables can detect ovulation-related physiological changes. These include wrist-worn devices like Ava [24], an in-ear thermometer [25], vaginal biosensors [26,27], and Oura Ring [28]. While temperature remains the primary continuously measured physiological metric for ovulation detection, other metrics such as heart rate, breath rate, and heart rate variability have also shown predictive value [1]. Studies indicate that intraoral and ear-based temperature methods are generally less accurate for ovulation detection compared with wrist and ring-based wearables [29], which provide more stable temperature readings, especially when measuring distal temperature during sleep [6,24,30]. The primary objective of these wearable technologies, thus far, has been to support conception or offer nonhormonal contraception by using changes in ovulation-related physiology to identify fertile days in a user’s cycle.

We assess the performance of the physiology method using Oura Ring of ovulation estimation against the traditional calendar method [31]. We hypothesized that the physiology method would outperform the calendar method in participants with high cycle variability, and the physiology method’s ovulation detection rate would decrease with participant age, reflecting age-related declines in progesterone levels and consequently luteal temperature rise magnitude [32]. Ultimately, we aim to review the strengths, weaknesses, and limitations of using Oura Ring physiology for ovulation date estimation.

## Methods

### Reference Ovulation Dates

Oura Ring commercial users can choose to self-report the results of home luteinizing hormone (LH) tests through the Oura Ring app. Positive LH test results served as the benchmark reference for algorithm performance testing. The reference ovulation date was defined as the day after the last positive LH test in the menstrual cycle [19,33].

### Data Inclusion and Exclusion Criteria

Participants were selected for the study if they self-reported a positive LH test result between January 1, 2019, and April 15, 2024. Inclusion criteria required the positive LH test date to be logged within a complete menstrual cycle, such that menses start and end dates were both logged. The self-reported menses and reference LH test dates were required to demarcate biologically plausible cycle phase lengths. Acceptable ranges were 10-90 days for the follicular phase and 8-20 days for the luteal phase [33]. If a single user had multiple complete menstrual cycles containing an LH test reference, these were included as long as the algorithm input window did not overlap. These inclusion criteria resulted in 121 users with 2 or more observations, for a total of 1209 ovulatory menstrual cycles from 1051 unique users.

We excluded menstrual cycles from the analysis based on insufficient physiology data, hormone use, or self-reported pregnancy. Insufficient data were defined as more than 40% of missing physiology data in the last 60 days. Hormone use was self-reported by a questionnaire, where participants were asked to report any hormone intake that could influence ovulation, including hormonal birth control, hormone replacement therapy, or fertility medications. Of these exclusion criteria, 47 cycles were excluded due to insufficient physiology data, 6 due to hormone use, and 1 due to pregnancy. The resulting dataset contained 1155 ovulatory cycles from 1007 unique participants.

### Ovulation Detection Algorithms

#### Calendar Method

We estimated each cycle’s ovulation date using the calendar method as a comparison with the physiology method results. The calendar method estimates the ovulation date by subtracting the typical luteal length from the user’s typical cycle length. The population mean is used as the typical luteal length, that is, 12 days [33,34]. One additional day is subtracted because we define the ovulation date as the last follicular day, which is standard in the literature [33,34]. The user’s typical cycle length is defined here as the median cycle length across the last 6 months, with outliers shorter than 12 days or greater than 90 days excluded.

#### Physiology Method

The physiology method is an algorithm written in Python that uses signal processing techniques to analyze continuously recorded finger temperature from Oura Ring to estimate the date of the most recent ovulation event. The ring contains negative temperature coefficient thermistors, which are used to measure skin temperature. Users are instructed to wear Oura Ring on a finger where the ring feels tight but not uncomfortable.

The algorithm was developed by assessing a separate training dataset of 30,000 menstrual cycles, which contained no overlapping users or menstrual cycles as the test set presented here. The algorithm aims to identify a maintained rise in skin temperature by around 0.3-0.7 °C, which has shown to be a robust postovulatory change following the regression of the dominant follicle [32]. The algorithm was tuned using a grid search across a set of parameters on the training dataset to optimize for detecting the rise in temperature following ovulation, as determined based on visual inspection. The physiology algorithm first normalizes the dataset by centering it around 0. Then, outliers were rejected, defined as >2 SD from the population average. Any missing or rejected data were then imputed using a linear fill. A Butterworth bandpass filter was then applied, with the low pass, high pass, and order set as parameters to be tuned in the grid search. Finally, hysteresis thresholding was used to determine the likely follicular and luteal phase days of the cycle, the parameters of which were also tuned in the grid search.

**Figure 1:**
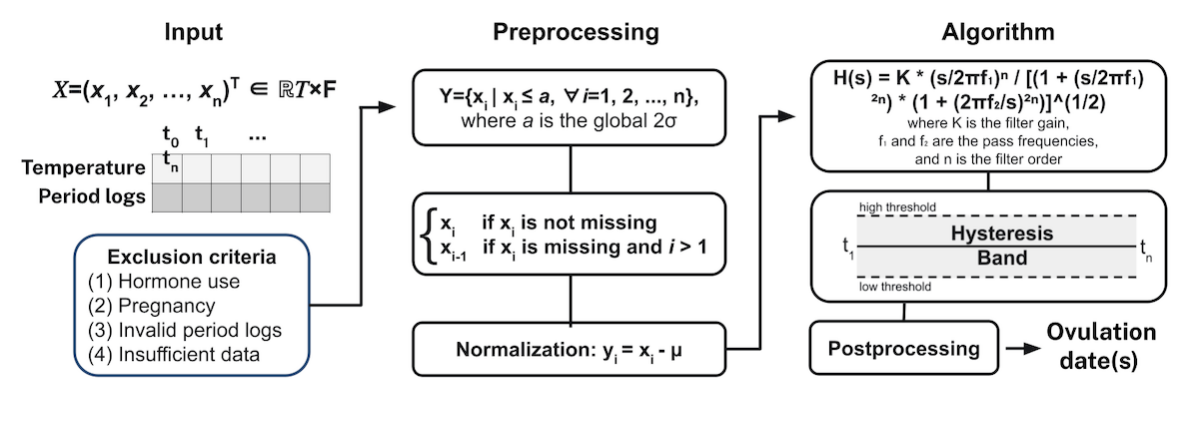
The physiology algorithm pipeline.

Algorithm postprocessing included combining the temperature-estimated luteal phase with self-reported period start logs. The algorithm rejects ovulation detections if they resulted in biologically implausible phase lengths [33,35], that is, luteal phases outside the range of 7-17 days or follicular phases outside the range of 10-90 days. In these cases, the physiology algorithm labels these cases as failures to detect ovulation. For details, refer to Figure 1.

### Statistical Analysis

#### Detection Rate

The ovulation detection rate was defined as the proportion of ovulatory cycles in which the algorithm correctly identified an ovulation. We calculated the 95% CI for the detection rate using the *z*-score–based margin of error for proportions [36]. To assess statistical differences in the ovulation detection rate across subgroups, we used the Fisher exact test. This nonparametric statistical method is well-suited for comparing proportion data, especially in datasets where subgroups vary in sample size [37]. For each subgroup of interest, such as cycle variation, we designated a reference group and used the Fisher exact test to compare the detection rates between the reference group with all other groups. For example, participants with irregular cycles were compared with participants with regular cycles as a reference group. Adjustments for multiple comparisons were made using the Bonferroni correction [38].

#### Accuracy

The ovulation date error was defined as the number of days between the algorithm’s estimated ovulation date and the reference ovulation date. The reference ovulation date was determined as the day following the last positive or peak LH surge reported in that cycle. To quantify the typical accuracy, we calculated the mean absolute error (MAE). The 95% CI on this mean was estimated using 1.96 times the SEM [39]. To evaluate statistical differences in ovulation date error across various subgroups, we used the Mann-Whitney *U* test. This test is preferred for providing a robust analysis with minimal influence from outliers and nonnormal distributions, in contrast to parametric methods. For each subgroup of interest, such as cycle variation, one group is designated as the reference group to be compared against all other groups. For example, we compare participants with irregular cycles to participants with regular cycles as a reference group. Adjustments for multiple comparisons were made using a Bonferroni correction [38].

### Subgroups

#### Cycle Length

Detection rate and accuracy were broken down by cycle length to compare ovulation estimation performance in menstrual cycles of various lengths. Cycle length was defined as the number of days between 2 menses onsets. The cycle length was classified as abnormal if it was shorter than 21 days or longer than 35 days [40]. Of the remaining cycle lengths, we defined a “short” cycle as lasting between 21-26 days, a “typical” cycle as lasting between 27-31 days, and a “long” cycle as lasting between 32-35 days (Table 1). To statistically compare ovulation detection performance metrics across groups, we compared performance metrics in cycles of typical length with all other cycle length groups.

**Table 1:** Cycle length.

Cycle length	Days, n	Cycles (n=1155), n (%)	Physiology method				Calendar method	
			Detection rate (%), mean (SEM)	MAE^a^, mean (SEM)	Within 2 days (%)	MAE, mean (SEM)		Within 2 days (%)
Abnormally short	≤21	2 (<1)	—^b^	—	—	—		—
Short	22-26	176 (15.2)	93.2 (3.7)	1.34 (.09)	84.8	2.84 (0.21)		51.7
Medium	27-31	662 (57.3)	98.0 (1.1)	1.18 (.04)	90	3.02 (0.13)		51
Long	32-35	223 (19.3)	97.3 (2.1)	1.26 (.08)	88	3.57 (0.28)		55.6
Abnormally long	≥36	92 (8)	90 (6)	1.70 (.18)	77	7.32 (1.07)		35

^a^MAE: mean absolute error.

^b^Not applicable.

#### Cycle Variability

Detection rate and accuracy were broken down by a participant’s cycle variability to compare performance in these groups. Cycle variability was defined as the median of the absolute value of cycle length differences between consecutive cycles, across the past 6 months. Abnormal cycle variation is defined as cycle variation larger than 7 days. Of the normal cycle variation, we defined a “typical” cycle variation as between 0-3 days and a “moderately irregular” cycle variation as between 4-7 days (Table 2). To statistically compare ovulation detection performance metrics across groups, we compared the typical cycle variability group with all other groups.

**Table 2:** Cycle variability.

Cycle variability	Variation (days)	Cycles (n=975), n (%)	Physiology method				Calendar method	
			Detection rate (%), mean (SEM)	MAE^a^, mean (SEM)	Within 2 days (%)	MAE, mean (SEM)		Within 2 days (%)
Typical	0-3	648 (67)	97.5 (1.2)	1.23 (0.05)	88.3	2.51 (0.08)		55.9
Moderately Irregular	4-7	210 (21)	96.0 (2.7)	1.23 (0.08)	90.7	3.48 (0.22)		43.8
Abnormally Irregular	>7	117 (12)	94.0 (4.3)	1.48 (0.14)	81.8	6.63 (0.65)		32.5

^a^MAE: mean absolute error.

#### Age

Age was binned into 4 groups. Specifically, our groups consisted of 18-26 years, 27-34 years, 35-43 years, and ≥44 years (Table 3). Because the group with the most participants fell into “27-34 years” age group, this was designated as the reference group for statistical analysis.

**Table 3:** Age.

Age group (years)	Cycles (n=1155), n (%)	Physiology method				Calendar method	
		Detection rate (%), mean (SEM)	MAE^a^, mean (SEM)	Within 2 days (%)	MAE, mean (SEM)		Within 2 days (%)
18-26	134 (11.6)	97.0 (2.8)	1.35 (0.11)	86.9	3.64 (0.48)		53
27-34	592 (51.3)	96.6 (1.5)	1.29 (0.05)	87.1	3.39 (0.48)		55.4
35-43	373 (32.3)	96.0 (2.0)	1.17 (0.06)	89.1	3.46 (0.21)		45
≥44	56 (5)	95 (6)	1.19 (0.13)	91	3.30 (0.31)		34

^a^MAE: mean absolute error.

**Ethical Considerations**

Data from adult Oura Ring users were selected from the Oura Ring commercial database. Oura Health Oy (Oulu, Finland) collected the data in accordance with their terms of use and privacy policy. By agreeing to the privacy policy, the Oura Ring users had also agreed to the use of their data to perform analysis. The data analysis study protocol was reviewed and approved by ŌURA’s science and legal team, which is responsible for ethics and regulatory adherence for studies. The study was deemed exempt as the analysis used aggregate data without individual identifiers. All data that were made available were deidentified and are anonymous. Additional protections included allowing data access only upon request for the purpose of ensuring the analyses presented are accurate and complete. Compensation for study participation was not provided.

## Results

### Participants

The study sample consisted of 964 female participants. Participants had a mean age of 32.8 (SD 5.5) years and a mean self-reported cycle length of 29.1 (SD 3.4) days.

### Algorithm Performance

**Figure 2:**
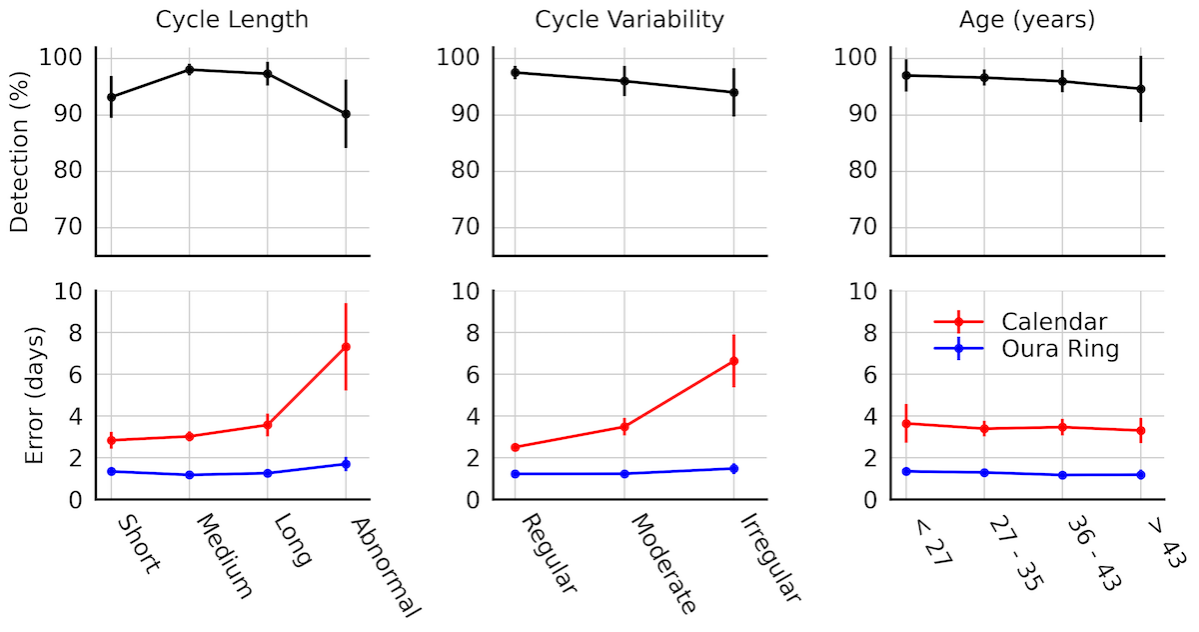
Ovulation estimation performance metrics across subgroups.

The physiology method from Oura Ring correctly detected 1113 (96.4%) of 1155 ovulatory cycles observed (95% CI 95.3-97.4%). The estimated ovulation date MAE was 1.26 (SEM 0.02) days. Of the total 1155 ovulatory cycles, 1097 (95%) of ovulations were detected within 3 days, 1015 (87.9%) were detected within 2 days, and 785 (68%) were detected within 1 day. By comparison, the calendar method’s ovulation MAE was 3.44 (SEM 0.07) days. In total, the calendar method detected 768 (66.5%) of ovulations within 3 days, 585 (50.7%) within 2 days, and 371 (32.2%) within 1 day. The physiology method estimated ovulation with significantly improved accuracy compared with the calendar method (*U*_1154_=904942, *P*<.001, Cliff *δ*=0.46). Refer to Figure 2 for a breakdown of the algorithm’s performance by cycle length, cycle variability, and age.

### Cycle Length

Ovulation detection rate was impacted by cycle length. The Fisher test for detection rates suggests the odds of detecting ovulation were 3.56 times higher in medium cycle lengths compared with short cycle lengths (odds ratio 3.56, 95% CI 1.65-8.06, *P*=.008). Long (*P*=.99) and abnormally long (*P*=.59) cycle lengths did not statistically differ relative to medium cycle lengths. Accuracy did not significantly differ across regular cycle lengths (short, medium, or long) when using the physiology method (short vs medium: *P*=.41; long vs medium: *P*=.99). However, abnormally long cycle lengths ≥36 days) were associated with increased error (*U*=22,383, *P*=.03, *δ*=−0.17) with an MAE of 1.7 (SEM .09) days compared with 1.18 (SEM .02) days. The calendar method also performed significantly worse in abnormally long cycles (*U*=21,184, *P*<.001, *δ*=−0.30), with an MAE of 7.32 (SEM .54) days compared with 3.02 (SEM .07) days.

### Cycle Variability

Neither detection rate nor accuracy differed significantly based on cycle variability when using the physiology method (for detection rate, moderate vs regular: *P*=.33 and irregular vs regular: *P*=.07; for accuracy, moderate vs regular: *P*=.99 and irregular vs regular: *P*=.15). However, the calendar method was associated with significantly worse accuracy for moderately irregular cyclers (*U*=54,093.5, *P*<.001, *δ*=−0.17) and even worse accuracy in irregular cycles (*U*=21,643, *P*<.001, *δ*=−0.43) when compared with typical cycle variability.

### Age

Neither the physiology method nor the calendar method was associated with differences across age in ovulation detection rate or estimated ovulation date error.

## Discussion

### Principal Findings

Finger temperature collected from Oura Ring was formulated as a physiology-based method of estimating the ovulation date. To assess the performance of this algorithm, we compare the physiology method with the calendar method in 1155 ovulatory cycles. The physiology method correctly detected ovulation in 1113 (96.4%) of 1155 cycles with an MAE of 1.26 days. This method was significantly more precise than the calendar method, which exhibited an MAE of 3.44 days, representing a 2.7-fold increase in error. In practical applications, Oura Ring primarily uses the physiology-based method to estimate a cycle’s ovulation date. However, the calendar method is used as a secondary approach when the physiology-based method cannot estimate the ovulation date, for example, in instances of excessive missing data. Thus, a thorough understanding of the strengths and limitations of both methods is critical. We also assessed if subgroups including age, cycle variation, and cycle length impact the accuracy of ovulation date estimation.

Age did not influence the accuracy of estimated ovulation dates within the assessed age range of 18-52 years, using either physiology-based or calendar methods. This finding aligns with the existing literature suggesting that age should not significantly affect the performance of the calendar method, as the luteal phase length remains consistent across age groups, and the calendar method estimates ovulation day by subtracting the luteal phase length from the user’s typical cycle length [31]. Although it was hypothesized that the physiology method may be less accurate across age due to variations in the magnitude of the postovulatory temperature rise [32,41], our analysis indicates that age did not significantly impact performance. However, ovulation estimation using Oura Ring has not yet been tested in individuals younger than 18 years and thus accuracy should not be assumed for adolescents.

Cycle variability significantly impacted the performance of the calendar method, but not the physiology method. Specifically, participants with irregular cycles experienced poorer performance using the calendar method, with an MAE of 6.63 days. This is a 2.6-fold decrease in accuracy relative to regular cyclers using the calendar method, and a 4.5-fold decrease in accuracy relative to the physiology-based method in irregular cyclers. When framing this as a window of days where the ovulation is 95% likely to have occurred, in irregular cyclers, that window is reduced from 14.2 days to 3.4 days. The superior accuracy of the physiology method likely stems from its independence from follicular phase length, which predominantly drives cycle variability and thus increases error in the calendar method [33,42].

Cycle length significantly influences ovulation estimation performance. Using the calendar method, longer cycles were associated with decreased accuracy, such that the MAE reaches 7.32 days in abnormally long cycles (ie, cycles exceeding 36 days in length). With the physiology method, statistical differences are evident both in the ovulation detection rate and the accuracy of the estimated ovulation date. Specifically, in cycles shorter than 26 days, which constitute 15% of our sample, the detection rate falls to 93%, compared with 98% in cycles of typical length. Furthermore, accuracy diminishes in abnormally long cycles with an MAE of 1.7 days versus 1.18 days in typical cycles. Consequently, users with shorter cycles may experience fewer detected ovulations, while those with abnormally long cycles may experience slightly worse accuracy when using the physiology method. Nevertheless, across all cycle categories, the physiology method significantly outperforms the calendar method.

### Comparison With Previous Work

Compared with wrist-worn devices with reported ovulation detection rates between 54% to 86% [43,44], Oura Ring exhibited superior performance, detecting 96.4% of ovulations. Oura Ring accuracy was similar to that of cervical mucus tracking, with 68% of estimations within 1 day of the reference ovulation date compared with 48% to 76% [19,20]. Oura Ring achieved favorable accuracy to BBT, which has a reported 20% detection rate [19,23], with an accuracy of 22% of ovulations correctly falling within 1 day of the reference ovulation [22,23]. Notably, BBT shows a marked decrease in performance in irregular cycles with a 34% drop in accuracy compared with regular cycles [45], while Oura Ring accuracy remained stable. Direct comparisons between studies should be approached with caution, as they are based on datasets with varying compositions of participant subgroups known to influence accuracy; notably, most studies exclude irregular cycles and cycles of abnormal length for performance analysis. The primary objective of this study is to identify conditions that may hinder the performance of Oura Ring ovulation estimation, rather than directly comparing it with other devices.

### Limitations

The primary limitation of this study is that our dataset consisted solely of ovulatory cycles and thus we cannot address ovulation estimation performance in anovulatory cycles. Without an estimate of the false alarm rate, a lack of detected ovulation by the algorithm should not be interpreted as indicative of an anovulatory cycle. The algorithm is not intended for use in individuals who frequently experience anovulatory cycles or who do not ovulate due to factors such as hormonal contraceptives.

Another limitation of this study is that our reference ovulation date is based on self-reported positive LH surge from participants using ovulation predictor kits (OPKs). While OPKs impose a lower participant burden, the gold standard for ovulation reference is a transvaginal ultrasound, which offers higher accuracy [19]. Given that our approach for collecting LH test results relied on self-report, the ovulation reference labels used here are susceptible to inaccuracies introduced by human error and subjective reporting biases. This approach also limits our sample to individuals who both use an Oura Ring and opt to take OPKs, and thus our cohort may not be representative of the broader population with menstrual cycles. Furthermore, the absence of data on negative OPK results precludes our ability to assess anovulatory cycles. In addition, it is not uncommon for an OPK to return a positive LH surge across multiple days; therefore, if participants logged only 1 positive LH surge and did not test on subsequent days, our reference ovulation day might be prematurely assigned.

A further limitation is that we did not collect data on whether participants had a history of menstrual cycle disorders. This is of particular concern in the 12% of participants with irregular cycles, who may have various underlying causes. A potential factor that could impact the accuracy of Oura Ring ovulation estimation is luteal phase deficiency (LPD), characterized by insufficient progesterone exposure [46]. LPD could influence the magnitude of the postovulatory temperature rise and thus impair the algorithm’s ability to estimate ovulation dates. Until future research is conducted, the estimated ovulation dates provided by the Oura Ring should be interpreted with caution in individuals with menstrual cycle disorders, particularly conditions associated with LPD, such as polycystic ovary syndrome [47,48], thyroid and prolactin disorders [49], and during in vitro fertilization cycles [50].

### Conclusions

Overall, the results suggest that Oura Ring offers significantly improved accuracy over the traditional calendar method for estimating ovulation date. Furthermore, the physiology method is associated with relatively robust accuracy across various ages, cycle variability, and cycle lengths, with only small drops in detecting rate for shorter cycles (<26 days) and slight drops in accuracy in abnormally long cycles (>36 days). This is significantly improved compared with the calendar method, which was associated with large accuracy decreases in users with long cycles and irregular cycles. Based on previous reports, Oura Ring ovulation estimation appears to be outperforming other wearables and BBT-based ovulation estimation methods and on par with the cervical-mucus tracking method, although those results should be compared cautiously given that the performance was measured on different datasets. Furthermore, we posit that widespread ovulation tracking could have broad use beyond its use in conception and nonhormonal contraception. Specifically, continuous monitoring of follicular and luteal phase lengths throughout the reproductive lifespan could benefit a wider demographic interested in menstrual cycle–related biomarkers. These biomarkers hold promise for the early detection of disorders related to the menstrual cycle and fertility.

## Abbreviations

BBT: basal body temperature
LH: luteinizing hormone
LPD: luteal phase deficiency
MAE: mean absolute error
OPK: ovulation predictor kit

## Data Availability

The dataset analyzed during this study is not publicly available due to privacy protections for participants but may be made available at Oura’s sole discretion.

## References

[1] Lyzwinski L, Elgendi M, Menon C (2024). Innovative approaches to menstruation and fertility tracking using wearable reproductive health technology: systematic review. J Med Internet Res.

[2] Yeh PT, Kennedy CE, van der Poel SV, Matsaseng T, Bernard L, Narasimhan M (2019). Should home-based ovulation predictor kits be offered as an additional approach for fertility management for women and couples desiring pregnancy? A systematic review and meta-analysis. BMJ Glob Health.

[3] Stanford JB, White GL, Hatasaka H (2002). Timing intercourse to achieve pregnancy: current evidence. Obstetrics & Gynecology.

[4] Robinson JE, Wakelin M, Ellis JE (2007). Increased pregnancy rate with use of the clearblue easy fertility monitor. Fertil Steril.

[5] Johnson S, Stanford JB, Warren G, Bond S, Bench-Capon S, Zinaman MJ (2020). Increased likelihood of pregnancy using an app-connected ovulation test system: a randomized controlled trial. J Womens Health (Larchmt).

[6] Pearson JT, Chelstowska M, Rowland SP, Benhar E, Kopp-Kallner H, Berglund Scherwitzl E, Acuna J, Gemzell Danielsson K, Scherwitzl R (2021). Contraceptive effectiveness of an FDA-cleared birth control app: results from the natural cycles U.S. cohort. J Womens Health (Larchmt).

[7] Freundl G, Sivin I, Batár I (2010). State-of-the-art of non-hormonal methods of contraception: IV. Natural family planning. Eur J Contracept Reprod Health Care.

[8] Jennings V, Haile LT, Simmons RG, Spieler J, Shattuck D (2019). Perfect- and typical-use effectiveness of the dot fertility app over 13 cycles: results from a prospective contraceptive effectiveness trial. Eur J Contracept Reprod Health Care.

[9] Watson A, Yarger J, Sedlander E, Urbina J, Hopkins K, Rodriguez MI, Fuentes L, Harper CC (2023). Concern that contraception affects future fertility: how common is this concern among young people and does it stop them from using contraception?. Contracept X.

[10] Schneider-Kamp A, Takhar J (2023). Interrogating the pill: rising distrust and the reshaping of health risk perceptions in the social media age. Soc Sci Med.

[11] Namasivayam V, Dehury B, Prakash R, Becker M, Anand P, Mishra A, Singhal S, Halli S, Blanchard J, Spears D, Isac S (2023). Understanding the rise in traditional contraceptive methods use in Uttar Pradesh, India. Reprod Health.

[12] le Guen M, Schantz C, Régnier-Loilier A, de la Rochebrochard E (2021). Reasons for rejecting hormonal contraception in western countries: a systematic review. Soc Sci Med.

[13] Symul L, Wac K, Hillard P, Salathé M (2019). Assessment of menstrual health status and evolution through mobile apps for fertility awareness. NPJ Digit Med.

[14] Crawford NM, Pritchard DA, Herring AH, Steiner AZ (2017). Prospective evaluation of luteal phase length and natural fertility. Fertil Steril.

[15] Harris BS, Steiner AZ, Faurot KR, Long A, Jukic AM (2023). Systemic inflammation and menstrual cycle length in a prospective cohort study. Am J Obstet Gynecol.

[16] Bakkensen JB, Christou G, Dimitriadis I, James K, Souter I (2020). The effect of follicular phase length on cycle outcomes and endometrial development in gonadotrophin ovarian stimulation/intrauterine insemination cycles. Reprod Biomed Online.

[17] Younis JS, Iskander R, Fauser BCJM, Izhaki I (2020). Does an association exist between menstrual cycle length within the normal range and ovarian reserve biomarkers during the reproductive years? A systematic review and meta-analysis. Hum Reprod Update.

[18] Stanford JB, Willis SK, Hatch EE, Rothman KJ, Wise LA (2019). Fecundability in relation to use of fertility awareness indicators in a North American preconception cohort study. Fertil Steril.

[19] Su HW, Yi YC, Wei TY, Chang TC, Cheng CM (2017). Detection of ovulation, a review of currently available methods. Bioeng Transl Med.

[20] Frank-Herrmann P, Heil J, Gnoth C, Toledo E, Baur S, Pyper C, Jenetzky E, Strowitzki T, Freundl G (2007). The effectiveness of a fertility awareness based method to avoid pregnancy in relation to a couple's sexual behaviour during the fertile time: a prospective longitudinal study. Hum Reprod.

[21] Händel P, Wahlström J (2019). Digital contraceptives based on basal body temperature measurements. Biomedical Signal Processing and Control.

[22] Barron ML, Fehring RJ (2005). Basal body temperature assessment: is it useful to couples seeking pregnancy?. MCN Am J Matern Child Nurs.

[23] Bauman JE (1981). Basal body temperature: unreliable method of ovulation detection. Fertil Steril.

[24] Goodale BM, Shilaih M, Falco L, Dammeier F, Hamvas G, Leeners B (2019). Wearable sensors reveal menses-driven changes in physiology and enable prediction of the fertile window: observational study. J Med Internet Res.

[25] Luo L, She X, Cao J, Zhang Y, Li Y, Song PXK (2020). Detection and prediction of ovulation from body temperature measured by an In-Ear wearable thermometer. IEEE Trans Biomed Eng.

[26] Aptekar D, Costantini L, Katilius J, Webster W (2016). Continuous, passive personal wearable sensor to predict ovulation 21G. Obstet Gynecol.

[27] Regidor PA, Kaczmarczyk M, Schiweck E, Goeckenjan-Festag M, Alexander H (2018). Identification and prediction of the fertile window with a new web-based medical device using a vaginal biosensor for measuring the circadian and circamensual core body temperature. Gynecol Endocrinol.

[28] Maijala A, Kinnunen H, Koskimäki H, Jämsä T, Kangas M (2019). Nocturnal finger skin temperature in menstrual cycle tracking: ambulatory pilot study using a wearable oura ring. BMC Womens Health.

[29] Baker FC, Siboza F, Fuller A (2020). Temperature regulation in women: effects of the menstrual cycle. Temperature (Austin).

[30] Alzueta E, de Zambotti M, Javitz H, Dulai T, Albinni B, Simon KC, Sattari N, Zhang J, Shuster A, Mednick SC, Baker FC (2022). Tracking sleep, temperature, heart rate, and daily symptoms across the menstrual cycle with the oura ring in healthy women. Int J Womens Health.

[31] Johnson S, Marriott L, Zinaman M (2018). Can apps and calendar methods predict ovulation with accuracy?. Curr Med Res Opin.

[32] Tatsumi T, Sampei M, Saito K, Honda Y, Okazaki Y, Arata N, Narumi K, Morisaki N, Ishikawa T, Narumi S (2020). Age-dependent and seasonal changes in menstrual cycle length and body temperature based on big data. Obstet Gynecol.

[33] Bull JR, Rowland SP, Scherwitzl EB, Scherwitzl R, Danielsson KG, Harper J (2019). Real-world menstrual cycle characteristics of more than 600,000 menstrual cycles. NPJ Digit Med.

[34] Najmabadi S, Schliep KC, Simonsen SE, Porucznik CA, Egger MJ, Stanford JB (2020). Menstrual bleeding, cycle length, and follicular and luteal phase lengths in women without known subfertility: a pooled analysis of three cohorts. Paediatr Perinat Epidemiol.

[35] Li H, Gibson EA, Jukic AMZ, Baird DD, Wilcox AJ, Curry CL, Fischer-Colbrie T, Onnela JP, Williams MA, Hauser R, Coull BA, Mahalingaiah S (2023). Menstrual cycle length variation by demographic characteristics from the apple women's health study. NPJ Digit Med.

[36] Agresti A, Coull BA (1998). Approximate is better than “Exact” for interval estimation of binomial proportions. The American Statistician.

[37] Kim HY (2017). Statistical notes for clinical researchers: chi-squared test and fisher's exact test. Restor Dent Endod.

[38] Armstrong RA (2014). When to use the bonferroni correction. Ophthalmic Physiol Opt.

[39] Altman DG, Bland JM (2005). Standard deviations and standard errors. BMJ.

[40] Attia GM, Alharbi OA, Aljohani RM (2023). The impact of irregular menstruation on health: a review of the literature. Cureus.

[41] Baerwald A, Vanden Brink H, Hunter C, Beuker D, Lim H, Lee C, Chizen D (2018). Age-related changes in luteal dynamics: preliminary associations with antral follicular dynamics and hormone production during the human menstrual cycle. Menopause.

[42] McKnight PE, Nujab J (2010). Mann-Whitney U Test.

[43] Shilaih M, Goodale BM, Falco L, Kübler F, de Clerck V, Leeners B (2018). Modern fertility awareness methods: wrist wearables capture the changes in temperature associated with the menstrual cycle. Biosci Rep.

[44] Zhu TY, Rothenbühler M, Hamvas G, Hofmann A, Welter J, Kahr M, Kimmich N, Shilaih M, Leeners B (2021). The accuracy of wrist skin temperature in detecting ovulation compared to basal body temperature: prospective comparative diagnostic accuracy study. J Med Internet Res.

[45] Yu JT, Su YF, Zhang C, Jin L, Lin XH, Chen LT, Huang HF, Wu YT (2022). Tracking of menstrual cycles and prediction of the fertile window via measurements of basal body temperature and heart rate as well as machine-learning algorithms. Reprod Biol Endocrinol.

[46] Palomba S, Santagni S, La Sala GB (2015). Progesterone administration for luteal phase deficiency in human reproduction: an old or new issue?. J Ovarian Res.

[47] Palomba S, Falbo A, Russo T, Orio F, Tolino A, Zullo F (2010). Systemic and local effects of metformin administration in patients with polycystic ovary syndrome (PCOS): relationship to the ovulatory response. Hum Reprod.

[48] Boutzios G, Karalaki M, Zapanti E (2013). Common pathophysiological mechanisms involved in luteal phase deficiency and polycystic ovary syndrome. impact on fertility. Endocrine.

[49] Daly DC, Walters CA, Soto-Albors CE, Riddick DH (1983). Endometrial biopsy during treatment of luteal phase defects is predictive of therapeutic outcome. Fertil Steril.

[50] Tavaniotou A, Albano C, Smitz J, Devroey P (2002). Impact of ovarian stimulation on corpus luteum function and embryonic implantation. J Reprod Immunol.

